# DMMR status and synchronous lesions predicts metachronous lesions after curative resection for rectal cancer

**DOI:** 10.3389/fsurg.2025.1510400

**Published:** 2025-01-21

**Authors:** Xijie Chen, Junguo Chen, Liang Xu, Dezheng Lin, Xiaoling Hong, Junsheng Peng, Xiaowen He, Jiancong Hu

**Affiliations:** ^1^ Guangdong Provincial Key Laboratory of Colorectal and Pelvic Floor Diseases, The Sixth Affiliated Hospital, Sun Yat-sen University, Guangzhou, Guangdong, China; ^2^ Department of General Surgery (Gastric Surgery), The Sixth Affiliated Hospital, Sun Yat-sen University, Guangzhou, Guangdong, China; ^3^ Biomedical Innovation Center, The Sixth Affiliated Hospital, Sun Yat-sen University, Guangzhou, Guangdong, China; ^4^ Department of Thoracic Surgery, Thoracic Cancer Center, The Sixth Affiliated Hospital, Sun Yat-sen University, Guangzhou, Guangdong, China; ^5^ Department of Pathology, The Sixth Affiliated Hospital, Sun Yat-sen University, Guangzhou, Guangdong, China; ^6^ Department of Endoscopic Surgery, The Sixth Affiliated Hospital, Sun Yat-sen University, Guangzhou, Guangdong, China; ^7^ Department of General Surgery (Colorectal Surgery), The Sixth Affiliated Hospital, Sun Yat-sen University, Guangzhou, Guangdong, China

**Keywords:** metachronous neoplasm, rectal cancer, deficient mismatch repair (dMMR), synchronous lesions, colonoscopy surveillance

## Abstract

**Background:**

There are no established standard colonoscopy surveillance protocols for patients after curative rectal cancer resection. We investigated the predictive factors for colorectal neoplasms during surveillance colonoscopies to identify patients who are at risk of developing metachronous neoplasms in the residual colorectum.

**Methods:**

This was a single-center, retrospective study that included patients with diagnosis of rectal carcinoma who had undergone curative resection from October 2012 to June 2018. Clinicopathological variables were analyzed by logistic regression analysis to identify risk factors independently associated with metachronous neoplasms in patients that underwent curative rectal cancer surgery.

**Results:**

In all, 554 patients were included in the analysis. Deficient mismatch repair (dMMR) status was recorded in 20 (3.6%) patients. At the surveillance colonoscopies, 118 patients (21.3%) had metachronous neoplasms while 169 patients (30.5%) had metachronous polyps. The median time interval between index colonoscopy and the last surveillance colonoscopy was 736.5 (476.75–1,082.25) days. Univariable and multivariable analysis showed dMMR status, synchronous adenomas/polyps, surveillance time > 3, and longer surveillance period patients were significant risk factors for development of metachronous lesions; in subgroup analysis, we also found that among rectal cancer patients with synchronous adenomas, adenomas located in the left colon and rectum, and longer surveillance period were independent risk factors for detecting metachronous adenomas.

**Conclusions:**

This study underscored the importance of extended follow-up protocols and targeted surveillance for identifying and managing metachronous lesions in dMMR rectal cancer patients, especially with synchronous adenomas. Further prospective, multicenter studies are needed to validate these results.

## Introduction

Colorectal cancer (CRC) is the third most commonly diagnosed cancer in the world (1). The prognosis for CRC has improved during the last few decades owing to the development of better diagnostic and treatment methods (2). The risk of developing metachronous adenoma is increased in patients who have undergone previous CRC curative resection (3), which indicated that postoperative surveillance is very important. A systematic review of endoscopic studies in the setting of post-CRC surgery showed the overall cumulative incidence of metachronous CRC was 2.2% (95% CI: 1.8%–2.9%) (4). Post-operative colonoscopy surveillance was indicated to prevent metachronous cancer to prolong survival. Previous studies have shown that despite the routine colonoscopy surveillance, interval cancer was still existed (5). Therefore, risk stratification based on risk factors of metachronous neoplasia is imperative.

A multicenter retrospective study was conducted to explore the risk factors for developing metachronous colorectal adenomas. Patients with a history of left-sided colon cancer had a significantly increased risk (6). In another study, synchronous advanced neoplasia instead of resection type was independently associated with the incidence of metachronous advanced neoplasia in patients after surgical resection of CRC (7). To our knowledge, no study yet has been conducted to explore the risk factors of developing metachronous neoplasia in rectal cancer patients after surgical resection. Rectal cancer is considerably different to colon cancer with respect to treatment and surveillance. After curative resection, nearly the entire colonic mucosa is preserved, which might still be risk for adenoma development.

The European Society of Gastrointestinal Endoscopy (ESGE) and European Society of Digestive Oncology (ESDO) guidelines recommend a high-quality perioperative colonoscopy before surgery for CRC or within 6 months following surgery; further, performing surveillance colonoscopy 1 year after CRC surgery was recommended (8). However, an intensive endoscopic surveillance strategy was not recommended given the lack of proven benefit. In patients with only rectal cancer, the American Gastroenterology Association has recommended flexible sigmoidoscopy every 3–6 months during first 2 years post-resection (9). No standard colonoscopy surveillance protocols were established, especially in patients after curative rectal cancer resection. An intensive strategy may be considered a waste of resources, and attempts to stratify the risk of metachronous neoplasms may result in more cost-effective strategies. The increasing demand in colonoscopy in patients after colorectal cancer curative resection was restricted by the shortage of colonoscopy resources. Especially because of the ongoing COVID-19 pandemic, unnecessary invasive examinations such as colonoscopy have been temporarily postponed (10).

The primary aim of the present study was to identify predictive factors of metachronous neoplasms in the residual colorectal mucosa at the surveillance colonoscopies in patients after curative rectal cancer surgery. We aimed to address the risk stratification of developing metachronous neoplasia after surgery using this approach, based on which a personalized colonoscopy surveillance strategy could be planned.

## Materials and methods

### Ethic statement

The authors are accountable for all aspects of the work in ensuring that questions related to the accuracy or integrity of any part of the work are appropriately investigated and resolved. This study was conducted in accordance with the ethical standards of the World Medical Association Declaration of Helsinki and the Ethical Guidelines for Clinical Research. Besides, the current study was approved by the Institutional Review Board of the Sixth Affiliated Hospital, Sun Yat-sen University.

### Inclusion and exclusion criteria

Consecutive patients with diagnosis of rectal carcinoma who had undergone curative resection from October 2012 to June 2018 were included in the study.

The inclusion criteria were: (i) pathological diagnosis of primary rectal cancer (with ICD-10 diagnosis of C20 in the medical record database); (ii) curative resection was performed; (iii) index colonoscopy report was available; and (iv) availability of reports of surveillance colonoscopies. The surveillance colonoscopies were performed completely to the cecum with appropriate bowel cleansing.

The index colonoscopy was defined as the perioperative colonoscopy or colonoscopy performed within 6 months after the surgical resection, completely to the cecum. For patients who were unable to undergo a full colorectal endoscopy preoperatively due to obstruction or other reasons, contrast-enhanced CT was routinely performed to exclude other synchronous lesions. Additionally, a postoperative colonoscopy was conducted within six months after surgery. The findings from the colonoscopy, combined with postoperative pathology results, were used to confirm the presence or absence of synchronous polyps and were considered as the index colonoscopy results. In cases where the index colonoscopy was performed after surgery, this colonoscopy was not considered as the first surveillance colonoscopy.

In accordance with the NCCN guidelines for the diagnosis and treatment of rectal cancer, we performed radical rectal cancer surgeries for patients who met the surgical indications. Specifically, anterior resection (AR) was performed for upper rectal cancer, low anterior resection (LAR) for mid-rectal cancer, and either abdominoperineal resection (APR) or ultra-low anterior resection (ULAR) for lower rectal cancer.

The exclusion criteria were: (i) patients with a diagnosis of familial adenomatous polyposis or inflammatory bowel disease); (ii) patients with diagnosis of multiple primary colorectal cancer with colon resection; (iii) patients with initial diagnosis of stage IV rectal cancer; and (iv) data available of only one surveillance colonoscopy.

### Data extraction

The following clinicopathological data were collected for each patient: gender; age at diagnosis; BMI, smoke, drink, hypertension, diabetes, family history of cancer, adjuvant chemotherapy and its regimen, bowel preparation, polyp number, adenoma number, synchronous advanced adenoma, neoadjuvant treatment, tumor location (upper, middle, or lower); cancer staging; mismatch repair gene (MMR) status; gene mutation status (*KRAS*, *NRAS*, *BRAF*, *PIK3CA*); synchronous adenomas or polyps at index colonoscopy; metachronous neoplasms or polyps at surveillance, surveillance time; and surveillance period (to surgery). Immunohistochemical (IHC) staining was performed for four proteins related to loss of expression of MMR genes (*MLH1*, *MSH2*, *MSH6*, and *PMS2*) and considered MMR deficiency (dMMR) if IHC staining was absent for any of them.

The primary endpoint of this study was the metachronous neoplasms (adenoma or cancer) at surveillance colonoscopies. The metachronous polyps data was also collected. It was defined as advanced if one of the following was applicable: adenomas ≥1 cm in size, tubulovillous or villous histology, and/or high-grade dysplasia (11).

### Statistical analysis

Continuous random variables and categorical variables were included in this study. In order to realize risk stratification of metachronous neoplasia, some continuous random variables were converted into binary or multi-categorical variables for data uniformity. One-sample Kolmogorov–Smirnov test was used to test the normality of distribution of continuous variables. Continuous variables with normal distribution were presented as mean [standard deviation (SD)], and non-normally distributed variables were presented as median [interquartile range (IQR)]. The categorical variables were reported as frequency [percentage (%)], and they were analyzed by chi-square test or Fisher's exact test as appropriate. Logistic stepwise regression was examined to figure out variables that were independently associated with metachronous neoplasia of patients after curative rectal cancer surgery. The degree of risk was interpreted by odds ratios (OR) with 95% confidence intervals (CI). Except for this, all other data were analyzed using the IBM SPSS statistics 26.0 (IBM corp., New York, USA). A two-sided *P* value of <0.05 was considered to indicate statistical significance.

## Results

### Study population and characteristics

**Figure 1:**
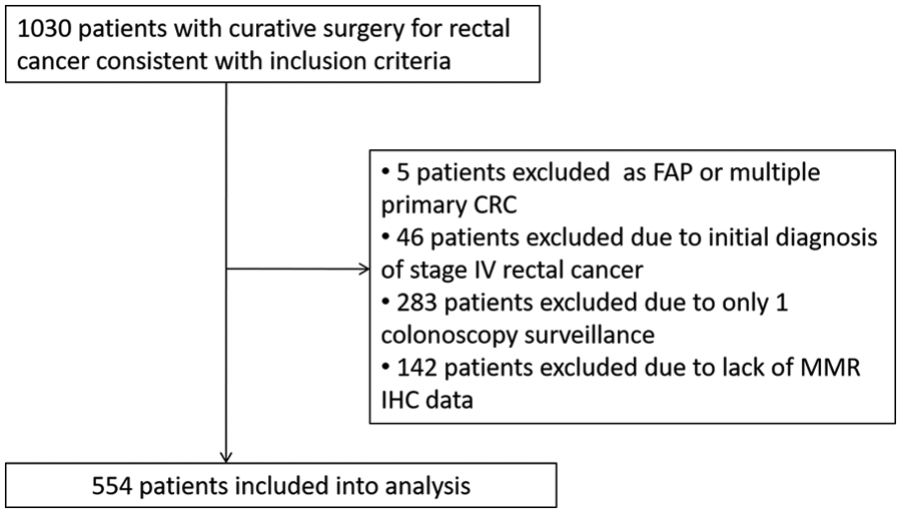
Flow chart of this study.

A total of 1,030 patients who underwent curative surgery for rectal cancer between October 2012 and June 2018 were included (Figure 1). All patients were offered participation in a scheduled follow up program. Five patients who were diagnosed with familial adenomatous polyposis or multiple primary colorectal cancer with colon resection were excluded. Forty-six patients with resectable metastatic lesions were excluded despite radical surgeries being performed. Further, 283 patients were excluded owing to only one surveillance colonoscopy, and 142 patients were excluded because of lack of MMR IHC data. Finally, 554 patients were included in the analysis.

The median time interval between index colonoscopy and the first surveillance colonoscopy was 191 days (124.75–263.25). 164 patients (29.6%) were younger than 50 years at diagnosis, and 200 patients (36.1%) were female. At index colonoscopy, 240 patients (43.3%) had synchronous polyps (100 patients with single polyp, 119 patients with multiple polyps), while 182 patients (32.9%) had synchronous adenomas (90 patients with single adenoma, 88 patients with multiple adenomas). Synchronous advanced adenomas were diagnosed in 80 (14.4%) patients at index colonoscopy; 225 patients (40.6%) accepted neoadjuvant therapy before surgery. Forty patients (7.2%) had low BMI (kg/m^2^) (BMI < 18.5), 193 patients (34.8%) had high BMI (BMI > 24). dMMR status was diagnosed in 20 (3.6%) patients (Table 1).

The median colonoscopy surveillance times was 3 (2–4). At the surveillance colonoscopies, 118 patients (21.3%) had metachronous neoplasms, while 169 patients (30.5%) had metachronous polyps. Two patients were diagnosed with interval cancer during surveillance. The median time interval between index colonoscopy and the last surveillance colonoscopy was 736.5 days (476.75–1,082.25).

### Identification of risk factors for metachronous polyps and metachronous neoplasm development

**Figure 2:**
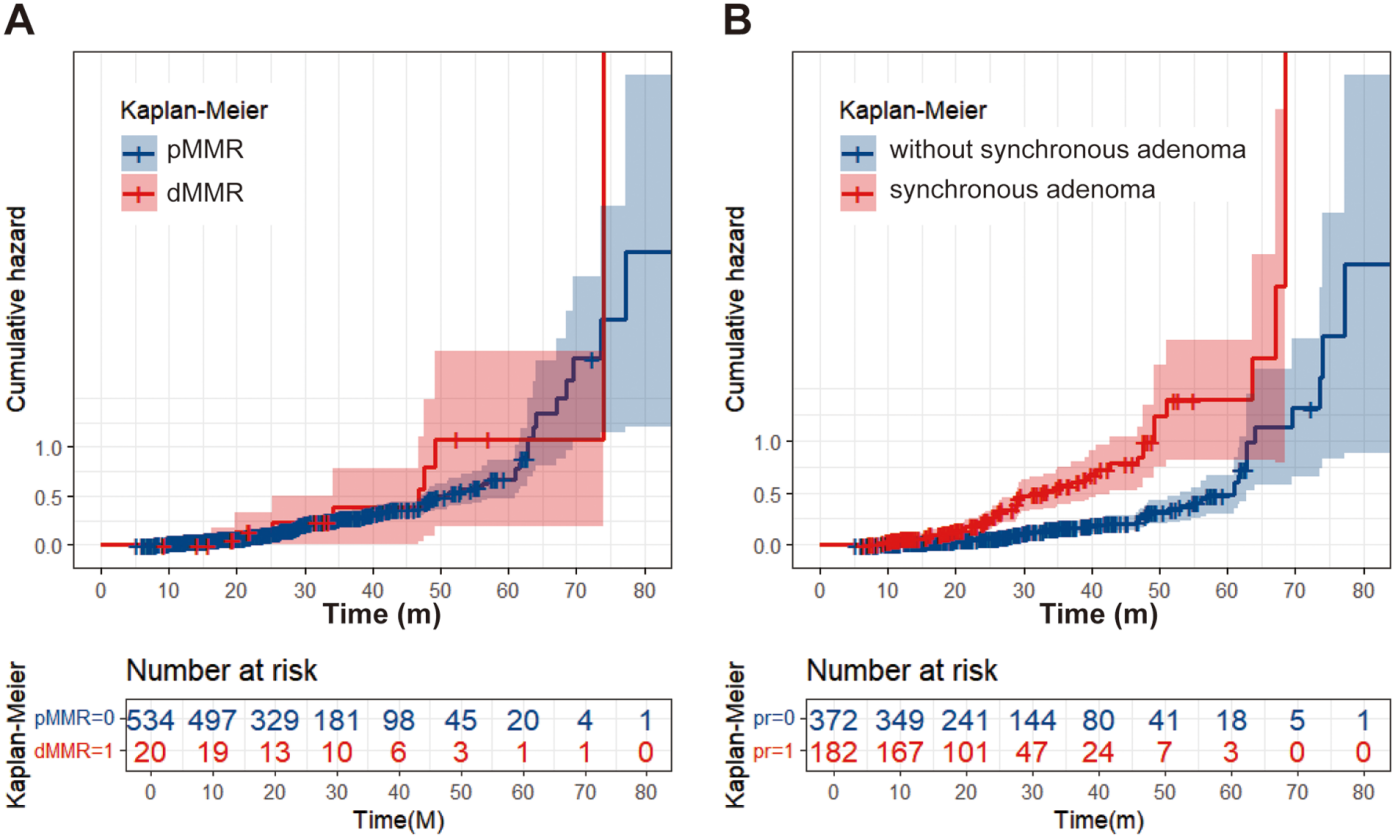
Cumulative incidence curve. **(A)** Patients with dMMR have a higher cumulative adenoma recurrence rate than patients with pMMR. **(B)** Patients with synchronous adenomas have a higher cumulative adenoma recurrence rate compared to patients without synchronous adenomas.

Univariable analysis indicated that dMMR, synchronous polyp, synchronous adenoma, surveillance times > 3, and longer surveillance period were positively correlated with metachronous polyps development. These factors were then put into multivariable analysis to find that dMMR (*P* = 0.012, OR = 3.455, 95% CI: 1.313–9.090), synchronous polyp (*P* < 0.0001, OR = 2.642, 95% CI: 1.762–3.907), surveillance times > 3 (*P* < 0.0001, OR = 2.195, 95% CI: 1.479–3.257), and longer surveillance period (*P* < 0.0001, OR = 1.032, 95% CI: 1.018–1.046) were independent risk factors for metachronous polyps development (Table 2). Likewise, multivariable analysis identified dMMR (*P* = 0.031, OR = 3.036, 95% CI: 1.049–1.108), synchronous adenoma (*P* < 0.0001, OR = 2.861, 95% CI: 1.825–4.484), surveillance times > 3 (*P* = 0.001, OR = 2.071, 95% CI: 1.328–3.228), and longer surveillance period (*P* < 0.0001, OR = 1.033, 95% CI: 1.018–1.049) as independent risk factors for metachronous neoplasm development (Table 1). As shown in Figure 2, rectal cancer patiens with dMMR status or synchronous adenomas have a higher cumulative incidence of metachronous adenomas after surgery.

**Table 1:** Demographic characteristics of rectal cancer patients and analysis of risk factors of metachronous neoplasm (MN) .

		Total	NMN	MN	Univariable analysis	Multivariable analysis	OR (95%CI)
					*P* value	*P* value	
Patients, *n* (%)		554 (100)	436 (78.7)	118 (21.3)			
Gender					0.762		
	Female	200 (36.1)	156 (35.8)	44 (37.3)			
	Male	354 (63.9)	280 (64.2)	74 (62.7)			
Age					0.638		
	<50	164 (29.6)	127 (29.1)	37 (31.4)			
	≥50	390 (70.4)	309 (70.9)	81 (68.6)			
BMI					0.547		
	≥18.5, <24	321 (57.9)	253 (58.0)	68 (57.6)			
	<18.5	40 (7.2)	34 (7.8)	6 (5.1)			
	≥24	193 (34.8)	149 (34.2)	44 (37.3)			
Smoke					0.596		
	No	490 (88.4)	384 (88.1)	106 (89.8)			
	Yes	64 (11.6)	52 (21.9)	12 (10.2)			
Drink					0.567		
	No	532 (95.8)	419 (96.1)	112 (94.9)			
	Yes	23 (4.2)	17 (3.9)	6 (5.1)			
Hypertension					0.476		
	No	476 (85.9)	377 (86.5)	99 (83.9)			
	Yes	78 (14.1)	59 (13.5)	19 (16.1)			
Diabetes					0.134		
	No	515 (93.0)	409 (93.8)	106 (89.8)			
	Yes	39 (7.0)	27 (6.2)	12 (10.2)			
Family history of cancer					0.885		
	No	534 (96.4)	420 (96.3)	114 (96.6)			
	Yes	20 (3.6)	16 (3.7)	4 (3.4)			
Adjuvant chemotherapy					0.212		
	No	212 (38.3)	161 (36.9)	51 (43.2)			
	Yes	342 (61.7)	275 (63.1)	67 (56.8)			
Bowel preparation					0.231		
	Excellent	28 (5.1)	20 (4.6)	8 (6.8)			
	Good	279 (50.4)	228 (52.3)	51 (43.2)			
	Poor	92 (16.6)	73 (16.7)	19 (16.1)			
	Inadequate	155 (28.0)	115 (26.4)	40 (33.9)			
Neoadjuvant tretment					0.283		
	No	329 (59.4)	264 (60.6)	65 (55.1)			
	Yes	225 (40.6)	172 (39.4)	53 (44.9)			
TNM stage					0.91		
	0	5 (0.9)	4 (0.9)	1 (0.8)			
	1	157 (28.3)	121 (27.8)	36 (30.6)			
	2	207 (37.4)	166 (38.1)	41 (34.7)			
	3	185 (33.4)	145 (33.2)	40 (33.9)			
Tumor location					0.312		
	Upper	135 (24.4)	105 (24.1)	30 (25.4)			
	Middle	206 (37.2)	169 (38.8)	37 (31.4)			
	Lower	213 (38.4)	162 (37.1)	51 (43.2)			
MMR					0.037	0.031	3.036 (1.049–1.108)
	pMMR	534 (96.4)	424 (97.2)	110 (93.2)			
	dMMR	20 (3.6)	12 (2.8)	8 (6.8)			
KRAS					0.884		
	Wild	430 (77.6)	339 (77.8)	91 (77.1)			
	Mutated	124 (22.4)	97 (22.2)	27 (22.9)			
NRAS					0.536		
	Wild	539 (97.3)	425 (97.5)	114 (96.6)			
	Mutated	15 (2.7)	11 (2.5)	4 (3.4)			
BRAF					0.116		
	Wild	551 (99.4)	435 (99.8)	116 (98.3)			
	Mutated	3 (0.6)	1 (0.2)	2 (1.7)			
PIK3CA					0.24		
	Wild	525 (94.4)	409 (93.8)	114 (96.6)			
	Mutated	31 (5.6)	27 (6.2)	4 (3.4)			
Adjuvant chemotherapy regimen					0.446		
	None	212 (38.3)	161 (36.9)	51 (43.2)			
	Single	44 (7.9)	36 (8.3)	8 (6.8)			
	Multiple	298 (53.8)	239 (54.3)	59 (50.0)			
Synchronous polyp					<0.0001		
	No	314 (56.7)	266 (61.0)	48 (40.7)			
	Yes	240 (43.3)	170 (39.0)	70 (59.3)			
Synchronous adenoma					<0.0001	<0.0001	2.861 (1.825–4.484)
	No	372 (67.1)	314 (72.0)	58 (49.2)			
	Yes	182 (32.9)	122 (28.0)	60 (50.8)			
Index colonoscopy time					0.065		
	Preoperative	221 (39.9)	355 (81.4)	87 (73.7)			
	Postoperation	333 (60.1)	81 (18.6)	31 (26.3)			
Surveillance time					<0.0001	0.001	2.071 (1.328–3.228)
	≤3	397 (71.7)	273 (62.6)	52 (44.1)			
	>3	157 (28.3)	163 (37.4)	66 (55.9)			
Surveillance period (to surgery)		24.4 (15.7,35.4)	23.5 (15.4,34.4)	28.4 (18.6,46.7)	0.002	<0.0001	1.033 (1.018–1.049)

**Table 2:** Demographic characteristics of rectal cancer patients and analysis of risk factors of metachronous polyp (MP).

		Total	NMP	MP	Univariable analysis	Multivariable analysis	OR (95%CI)
					*P* value	*P* value	
Patients, *n* (%)		554 (100)	385 (69.5)	169 (30.5)			
Gender					0.849		
	Female	200 (36.1)	138 (35.8)	62 (36.7)			
	Male	354 (63.9)	247 (64.2)	107 (63.3)			
Age					0.835		
	<50	164 (29.6)	115 (38.9)	49 (29.0)			
	≥50	390 (70.4)	270 (70.1)	120 (71.0)			
BMI					0.324		
	≥18.5, <24	321 (57.9)	226 (58.7)	95 (56.2)			
	<18.5	40 (7.2)	31 (8.1)	9 (5.3)			
	≥24	193 (34.8)	128 (33.2)	65 (38.5)			
Smoke					0.88		
	No	490 (88.4)	340 (88.3)	150 (27.1)			
	Yes	64 (11.6)	45 (11.7)	19 (3.4)			
Drink					0.359		
	No	531 (95.8)	371 (96.4)	160 (88.8)			
	Yes	23 (4.2)	14 (3.6)	9 (21.2)			
Hypertension					0.558		
	No	476 (85.9)	333 (86.5)	143 (84.6)			
	Yes	78 (14.1)	52 (13.5)	26 (15.4)			
Diabetes					0.263		
	No	515 (93.0)	361 (93.8)	154 (91.1)			
	Yes	39 (7.0)	24 (6.2)	15 (8.9)			
Family history of cancer					0.586		
	No	534 (96.4)	370 (96.1)	164 (97.0)			
	Yes	20 (3.6)	15 (3.9)	5 (3.0)			
Adjuvant chemotherapy					0.899		
	No	212 (38.3)	148 (38.4)	64 (37.9)			
	Yes	342 (61.7)	237 (61.6)	105 (62.1)			
Bowel preparation					0.497		
	Excellent	28 (5.1)	17 (4.4)	11 (6.5)			
	Good	279 (50.4)	200 (51.9)	79 (46.7)			
	Poor	92 (16.6)	65 (16.9)	27 (16.0)			
	Inadequate	155 (28.0)	103 (26.8)	52 (30.8)			
Neoadjuvant tretment					0.314		
	No	329 (59.4)	234 (60.8)	95 (56.2)			
	Yes	225 (40.6)	151 (39.2)	74 (43.8)			
TNM stage					0.958		
	0	5 (0.9)	4 (0.1)	1 (0.6)			
	1	157 (28.3)	110 (28.6)	47 (27.8)			
	2	207 (37.4)	143 (37.1)	64 (37.9)			
	3	185 (33.4)	128 (33.2)	57 (33.7)			
Tumor location					0.908		
	Upper	135 (24.4)	92 (23.9)	43 (25.4)			
	Middle	206 (37.2)	145 (37.7)	61 (36.1)			
	Lower	213 (38.4)	148 (38.4)	65 (38.5)			
MMR					0.015	0.012	3.455 (1.313–9.090)
	pMMR	534 (96.4)	376 (97.7)	158 (93.5)			
	dMMR	20 (3.6)	9 (2.3)	11 (6.5)			
KRAS					0.172		
	Wild	430 (77.6)	305 (79.2)	125 (74.0)			
	Mutated	124 (22.4)	80 (20.8)	44 (26)			
NRAS					0.782		
	Wild	539 (97.3)	375 (97.4)	164 (97.0)			
	Mutated	15 (2.7)	10 (2.6)	5 (3.0)			
BRAF					0.222		
	Wild	551 (99.4)	384 (99.7)	167 (98.8)			
	Mutated	3 (0.6)	1 (0.3)	2 (1.2)			
PIK3CA					0.324		
	Wild	523 (94.4)	361 (93.8)	162 (95.9)			
	Mutated	30 (5.6)	24 (6.2)	7 (4.1)			
Adjuvant chemotherapy regimen					0.465		
	None	212 (38.3)	148 (38.4)	64 (37.9)			
	Single	44 (7.9)	34 (8.8)	10 (5.9)			
	Multiple	298 (53.8)	203 (52.8)	95 (56.2)			
Synchronous polyp					<0.0001	<0.0001	2.642 (1.762–3.907)
	No	314 (56.7)	244 (63.4)	70 (41.4)			
	Yes	240 (43.3)	141 (36.6)	99 (58.6)			
Synchronous adenoma					<0.0001		
	No	372 (67.1)	280 (72.7)	92 (54.4)			
	Yes	182 (32.9)	105 (27.3)	77 (45.6)			
Index colonoscopy time					0.072		
	Preoperative	442 (79.8)	315 (81.8)	127 (75.1)			
	Postoperation	112 (20.2)	70 (18.2)	42 (24.9)			
Surveillance time					<0.0001	<0.0001	2.195 (1.479–3.257)
	≤3	325 (58.7)	248 (64.4)	77 (45.6)			
	>3	229(41.3)	137(35.6)	92(54.4)			
Surveillance period (to surgery)		24.4 (15.7,35.4)	22.6 (15.2,33.4)	26.8 (18.9,42.0)	0.001	<0.0001	1.032 (1.018–1.046)

### Subgroup analysis

In order to clarify the impact of synchronous lesions on metachronous lesions in patients with rectal cancer, we performed subgroup analysis based on the presence or absence of synchronous lesions. As shown in Table 3, there were a total of 182 patients with synchronous adenomas, among whom 61 patients had adenomas distributed in the right colon (with the splenic flexure of the colon as the boundary) and 121 patients had adenomas distributed in the left colon and rectum. Univariate and multivariate analyses revealed that synchronous adenomas distributed in the left colon and rectum, as well as longer surveillance period, were associated with a higher likelihood of detecting metachronous neoplasm. Moreover, among patients with synchronous polyps, dMMR status, surveillance times > 3, and longer surveillance period were associated with an increased likelihood of detecting metachronous polyps (Table 4).

**Table 3:** Demographic characteristics of rectal cancer patients with synchronous adenoma and analysis of risk factors of metachronous neoplasm (MN).

		Total	NMN	MN	Univariable analysis	Multivariable analysis	OR (95%CI)
					*P* value	*P* value	
Patients, *n* (%)		182 (100)	122 (67.0)	60 (33.0)			
Gender					0.462		
	Female	66 (36.3)	42 (34.4)	24 (40.0)			
	Male	116 (63.7)	80 (65.6)	36 (60.0)			
Age					0.679		
	<50	54 (29.7)	35 (28.7)	19 (31.7)			
	≥50	128 (70.3)	87 (71.3)	41 (68.3)			
BMI					0.252		
	≥18.5, <24	103 (56.6)	67 (54.9)	36 (60.0)			
	<18.5	14 (7.7)	12 (9.8)	2 (3.3)			
	≥24	65 (35.7)	43 (35.2)	22 (36.7)			
Smoke					1		
	No	167 (91.8)	112 (91.8)	55 (91.7)			
	Yes	15 (8.2)	10 (8.2)	5 (8.3)			
Drink					0.268		
	No	177 (97.3)	117 (95.9)	60 (100)			
	Yes	5 (2.7)	5 (4.1)	0 (0)			
Hypertension					0.184		
	No	160 (87.9)	110 (90.2)	50 (83.3)			
	Yes	22 (12.1)	12 (9.8)	10 (16.7)			
Diabetes					0.305		
	No	161 (88.5)	110 (90.2)	51 (85.0)			
	Yes	21 (11.5)	12 (9.8)	9 (15.0)			
Family history of cancer					1		
	No	177 (97.3)	119 (97.5)	58 (96.7)			
	Yes	5 (2.7)	3 (2.5)	2 (3.3)			
Adjuvant chemotherapy					0.09		
	No	72 (39.6)	43 (35.2)	29 (48.3)			
	Yes	110 (54.9)	79 (64.8)	31 (51.7)			
Bowel preparation					0.517		
	Excellent	9 (4.9)	4 (2.2)	5 (8.3)			
	Good	93 (51.1)	64 (52.5)	29 (48.3)			
	Poor	28 (15.4)	18 (14.8)	10 (16.7)			
	Inadequate	52 (28.6)	36 (29.5)	16 (26.7)			
Neoadjuvant tretment					0.249		
	No	105 (57.7)	74 (60.7)	31 (51.7)			
	Yes	77 (42.3)	48 (39.3)	29 (48.3)			
TNM stage					0.348		
	0	1 (0.6)	0 (0)	1 (1.6)			
	1	51 (28.0)	32 (26.2)	19 (31.7)			
	2	67 (36.8)	48 (39.3)	19 (31.7)			
	3	63 (34.6)	42 (34.4)	21 (35.0)			
Tumor location					0.562		
	Upper	45 (24.7)	31 (30.2)	14 (23.3)			
	Middle	68 (37.4)	48 (39.3)	20 (33.3)			
	Lower	69 (37.9)	43 (35.2)	26 (43.4)			
MMR					0.069		
	pMMR	174 (95.6)	119 (97.5)	55 (91.7)			
	dMMR	8 (4.4)	3 (2.5)	5 (8.3)			
KRAS					0.662		
	Wild	139 (76.4)	92 (75.4)	47 (78.3)			
	Mutated	43 (23.6)	30 (24.6)	13 (21.7)			
NRAS					0.886		
	Wild	177 (97.3)	118 (96.7)	59 (98.3)			
	Mutated	5 (2.7)	4 (3.3)	1 (1.7)			
BRAF					0.716		
	Wild	181 (99.5)	122 (1.0)	59 (98.3)			
	Mutated	1 (0.5)	0 (0)	1 (1.7)			
PIK3CA					0.159		
	Wild	171 (94.0)	112 (91.8)	59 (98.3)			
	Mutated	11 (6.0)	10 (8.2)	1 (1.7)			
Adjuvant chemotherapy regimen					0.24		
	None	72 (39.6)	43 (35.2)	29 (48.3)			
	Single	14 (7.7)	10 (8.2)	4 (6.7)			
	Multiple	90 (52.7)	69 (56.6)	27 (45.0)			
Adenoma location					0.018	0.02	2.362 (1.146–4.867)
	Proximal colon	61 (33.5)	48 (39.3)	13 (21.7)			
	Distal colon	121 (66.5)	74 (60.1)	47 (78.3)			
Index colonoscopy time					0.352		
	Preoperative	155 (85.2)	106 (86.9)	49 (81.7)			
	Postoperation	27 (14.8)	16 (13.1)	11 (18.3)			
Surveillance time					0.02		
	≤3	86 (47.3)	65 (53.3)	21 (35.0)			
	>3	96 (52.7)	57 (46.7)	39 (65.0)			
Surveillance period (to surgery)		22.4 (13.3, 31.6)	19.8 (12.8, 29.2)	25.4 (12.8, 29.2)	0.048	0.031	1.028 (1.003–1.054)

**Table 4:** Demographic characteristics of rectal cancer patients with synchronous polyps and analysis of risk factors of metachronous polyps (MP).

		Total	NMP	MP	Univariable analysis	Multivariable analysis	OR (95%CI)
					*P* value	*P* value	
Patients, *n* (%)		240 (100)	141 (58.8)	99 (41.2)			
Gender					0.627		
	Female	83 (34.6)	47 (33.3)	36 (36.4)			
	Male	157 (65.4)	94 (66.7)	63 (63.6)			
Age					0.781		
	<50	80 (33.3)	46 (32.6)	34 (34.3)			
	≥50	160 (66.7)	95 (67.4)	65 (65.7)			
BMI					0.385		
	≥18.5, <24	137 (57.1)	81 (57.4)	56 (56.6)			
	<18.5	21 (8.8)	15 (10.6)	6 (6.1)			
	≥24	82 (34.2)	45 (31.9)	37 (37.4)			
Smoke					0.828		
	No	217 (90.4)	127 (90.1)	90 (90.9)			
	Yes	23 (9.6)	14 (9.9)	9 (9.1)			
Drink					0.559		
	No	232 (96.7)	135 (95.7)	97 (98.0)			
	Yes	8 (3.3)	6 (4.3)	2 (2.0)			
Hypertension					0.222		
	No	211 (87.9)	127 (90.1)	84 (84.8)			
	Yes	29 (12.1)	14 (9.9)	15 (15.2)			
Diabetes					0.359		
	No	216 (90.0)	129 (91.5)	87 (87.9)			
	Yes	24 (10.0)	12 (8.5)	12 (12.1)			
Family history of cancer					1		
	No	233 (97.1)	137 (97.2)	96 (97.0)			
	Yes	7 (2.9)	4 (2.8)	3 (3.0)			
Adjuvant chemotherapy					0.268		
	No	99 (41.3)	54 (38.4)	45 (45.4)			
	Yes	141 (58.8)	87 (61.7)	54 (54.5)			
Bowel preparation					0.47		
	Excellent	11 (4.6)	4 (2.8)	7 (7.1)			
	Good	118 (49.2)	72 (51.1)	46 (46.4)			
	Poor	35 (14.6)	20 (14.2)	15 (15.2)			
	Inadequate	76 (31.7)	45 (31.9)	31 (31.3)			
Neoadjuvant tretment					0.119		
	No	145 (60.4)	91 (64.5)	54 (54.5)			
	Yes	95 (39.6)	50 (35.5)	45 (45.4)			
TNM stage					0.95		
	0	2 (0.9)	1 (0.8)	1 (3.1)			
	1	66 (27.5)	37 (26.2)	29 (27.2)			
	2	92 (38.3)	55 (39.0)	37 (37.4)			
	3	80 (33.3)	48 (34.0)	32 (32.3)			
Tumor location					0.74		
	Upper	57 (23.8)	36 (25.5)	21 (21.2)			
	Middle	92 (38.3)	53 (37.6)	39 (39.4)			
	Lower	91 (37.9)	52 (36.9)	39 (39.4)			
MMR					0.063	0.004	3.92 (1.528–10.059)
	pMMR	229 (95.4)	138 (97.9)	91 (91.9)			
	dMMR	11 (4.6)	3 (2.1)	8 (8.1)			
KRAS					0.644		
	Wild	188 (78.3)	109 (77.3)	79 (79.8)			
	Mutated	52 (21.7)	32 (22.7)	20 (20.2)			
NRAS					0.606		
	Wild	235 (97.9)	137（97.2）	98 (99.0)			
	Mutated	5 (2.1)	4 (2.8）	1 (1.0）			
BRAF					0.859		
	Wild	239 (99.6)	141 (100)	98 (99.0)			
	Mutated	1 (0.4)	0 (0)	1 (1.0）			
PIK3CA					0.121		
	Wild	226 (94.2)	130 (92.2)	96 (97.0)			
	Mutated	14 (5.8)	11 (7.6)	3 (3.0)			
Adjuvant chemotherapy regimen					0.484		
	None	99 (41.3)	54 (38.3)	45 (45.5)			
	Single	16 (6.7)	9 (6.4)	7 (7.1)			
	Multiple	125 (52.1)	78 (55.3)	47 (47.5)			
Polyp location					0.244		
	Proximal colon	80 (33.3)	51 (36.2)	29 (29.0)			
	Distal colon	160 (66.7)	90 (63.8)	70 (71.0)			
Synchronous adenoma					0.555		
	No	58 (24.2)	36 (25.5)	22 (22.2)			
	Yes	182 (75.8)	105 (74.5)	77 (77.8)			
Index colonoscopy time					0.404		
	Preoperative	121 (85.8)	121 (85.8)	81 (81.3)			
	Postoperation	38 (15.8)	20 (14.2)	18 (18.2)			
Surveillance time					0.009	0.001	2.454 (1.671–3.605)
	≤3	121 (50.4)	81 (57.4)	40 (40.4)			
	>3	119(49.6)	60(42.6)	59(59.6)			
Surveillance period (to surgery)		22.4 (13.7,32.5)	19.6 (12.9,29.7)	25.4 (16.0,36.0)	0.011	0.001	1.001 (1.000–1.001)

## Discussion

In this study, we found that dMMR status, synchronous adenoma, surveillance time > 3, and longer surveillance period were significantly more likely to develop metachronous neoplasm at the surveillance colonoscopy than others. This is consistent with the risk factors for metachronous polyps in rectal cancer patients with synchronous polyps. Furthermore, in subgroup analysis, we also found that among rectal cancer patients with synchronous adenomas, adenomas located in the left colon and rectum, and longer surveillance period were independent risk factors for detecting metachronous adenomas.

Colorectal cancer (CRC) surgery is widely recognized as a procedure with significant trauma and a high risk of postoperative complications, among which surgical site infection (SSI) is the most common. SSI causes substantial pain and suffering to patients and is associated with increased healthcare costs, morbidity, prolonged hospital stays, readmissions, sepsis, and mortality (12). Reports indicate that sepsis due to SSI occurs in >1% of elective surgeries and >4% of emergency surgeries, primarily due to inadequate bowel preparation, anastomotic tension leading to leakage, and patient comorbidities. The mortality rate for postoperative sepsis approaches 25%, with patients having recurrent/metastatic CRC at even higher risk for SSI. Novel enzymes, such as butyrylcholinesterase (13), have been proposed as biomarkers to predict these complications, although further clinical validation is needed. To reduce these risks, patient risk stratification and personalized colonoscopy follow-up strategies are essential. Recently, deep learning (DL) algorithms have demonstrated efficacy in improving CRC detection rates (14) and enabling accurate histological classification (15). The integration of colonoscopy with DL algorithms holds promise for preventing disease progression at earlier stages.

To our knowledge, no standard colonoscopy surveillance protocol has been established in patients after curative rectal cancer resection. The first surveillance colonoscopy was recommended in the first year after CRC surgery (8). Neoadjuvant therapy strategy and different risk of anastomosis leakage and stenosis in rectal cancer treatment made the post-operative follow-up quite different when compared to patients with colon cancer (16). In our center, patients with temporary stoma after curative rectal cancer resection accepted the first colonoscopy within 3 months. The American Gastroenterology Association recommends flexible sigmoidoscopy every 3–6 months during the first 2 years post-resection in patients with rectal cancer (9). It was assumed that in patients who undergo only single colonoscopy surveillance, the true situation of actual metachronous neoplasms may not be reflected. To avoid this potential bias, patients with only one colonoscopy surveillance were excluded in our study.

Several studies focused on the risk factors of development of metachronous adenoma of colorectal cancer. Most studies included only colon cancer patients; in that, synchronous adenoma and left-sided colectomy were independent predictors of adenoma detection on surveillance colonoscopy (6, 17, 18). Two studies included both colon and rectal cancer patients with a controversial conclusion about the relationship of tumor site and the risk of metachronous adenoma development (7, 19). Generally, rectal cancer is very different from colon cancer with respect to treatment strategy and surveillance (20). In the present study, we provided a large population of rectal cancer after curative surgery with detailed colonoscopy surveillance. In our study, the risk of metachronous neoplasm/polyps increased in patients with synchronous adenoma or polyps. This was consistent with previous colorectal studies (21, 22). However, in our study, advanced age, diabetes, and male gender did not show statistically significant differences in terms of increased risk of developing metachronous lesions. This may be attributed to our study's inclusion criteria limited to rectal cancer patients only. We are aware that adenomatous polyps in the rectal region have a higher recurrence rate, and factors such as age, diabetes, and gender do not play a significant predictive role in adenoma recurrence. Therefore, in a limited population and a short follow-up period, it may be difficult to identify clear differences. It is worth noting that our study also concluded that more frequent follow-up visits and longer surveillance period are predictive factors for detecting metachronous adenomas, which is consistent with previous research findings (17, 23).

In order to explore personalized follow-up strategies, we also investigated the impact of the initial polyp or adenoma location on metachronous lesions. Our study demonstrated that adenomas found during the initial colonoscopy in the left colon and rectum were more likely to develop metachronous adenomas. This finding is consistent with the conclusion mentioned in the study by Chunmei Guo et al. (24), which identified right-sided colon cancer as an independent risk factor for metachronous adenomas. However, a study by Kwangwoo Nam et al. (21) reported that distal colon cancer (splenic flexure and below) had over four times the risk of metachronous advanced neoplasia during follow-up compared to proximal lesions, which differs from our study's conclusion. This discrepancy may be explained as follows. Firstly, adenomas in the right colon are more prone to being overlooked, so endoscopists in our center consciously increase observation time for lesions in the right colon during examinations to enhance adenoma clearance rates. Secondly, rectal polyps themselves have a higher recurrence rate compared to other locations. However, colon cancer and rectal cancer are distinct entities, and drawing conclusions solely from studies on colon cancer patients is evidently insufficient. Further studies with larger sample sizes are needed to validate this conclusion.

Mismatch repair deficiency was found in approximately 10%–15% of CRC (25, 26), which can be assessed on the basis of microsatellite instability or loss-of-expression of MMR proteins (27). MMR deficiency is often detected by IHC in cancer tissue specimens in clinical practice. dMMR CRC is often secondary to Lynch syndrome. Monoallelic pathogenic germline mutations in MMR pathway genes (*MLH1*, *MSH2*, *MSH6*, *PMS2*, *EPCAM*) can be detected in patients with Lynch syndrome (28). MMR deficiency can also occur as a sporadic (non-hereditary) process. The sporadic process was characterized by a distinctive hyper-proliferative, serrated morphology, with DNA methylation abnormalities (26). Previous studies showed that Lynch syndrome manifests as a predominantly right-sided colon cancer with a propensity for synchronous and metachronous colorectal cancers (28, 29). Few studies have focused on dMMR rectal cancer. In the present study, dMMR status was an independent risk factor in the prediction of metachronous adenoma development in patients after curative rectal cancer surgery. A retrospective study showed that patients meeting the Amsterdam criteria for diagnosis of hereditary nonpolyposis colorectal cancer and undergoing partial colectomy had a high rate of metachronous high-risk adenomas and carcinomas (30). In another retrospective cohort study, the risk of metachronous colorectal cancer among patients with MMR gene mutations was 16% at 10 years, which increased to 62% at 30 years (31). Colectomy with ileorectal anastomosis as the primary procedure for the treatment was recommended in CRC patients with Lynch syndrome in the Guidelines of the U.S. Multi-Society Task Force on Colorectal Cancer (32). The majority of CRC patients were treated before the genetic counseling or testing in practice. The extensive colectomy was not widely accepted especially in rectal cancer patients. The guidelines recommend endoscopic surveillance of the residual rectum after sub-total colectomy every 6 or 12 months in patients with Lynch syndrome (32). Furthermore, patients with advanced-stage dMMR rectal cancer appear to exhibit significant sensitivity to PD-1 inhibitors. A study conducted by Cercek et al. (33) reported that all 14 patients treated with PD-1 inhibitors achieved complete clinical response (cCR), with no instances of recurrence or progression observed during a follow-up period ranging from 6 to 25 months. Another study (34) reporting on the long-term efficacy of cCR patients demonstrated a three-year disease-free survival rate and overall survival rate of 100%. Notably, in the PICC study (35), PD-1 inhibitor monotherapy administered as neoadjuvant treatment to chemotherapy-insensitive patients resulted in a cCR rate exceeding 65%. Despite the favorable outcomes observed, our research findings indicate that these patients still face a heightened risk of adenoma recurrence. Therefore, in a “watch and wait” approach, intensive colonoscopy surveillance should be implemented. Taking our present results together, an intensive surveillance strategy may be considered in dMMR patients after rectal cancer curative resection.

This study should be regarded as an initial exploration in terms of the risk factors of metachronous neoplasm development in patients after rectal cancer curative resection. However, caution should be exercised when interpreting the findings due to some study limitations. First, this is a retrospective study with some missing data. Patients without IHC data were excluded. However, the present study population is largely focused only on rectal cancer patients with at least three colonoscopy examinations conducted at the same center. Second, some patients did not have pre-operation colonoscopy data or full colorectal colonoscopy data due to obstruction. We checked the pre-operation colonoscopy data in other centers and considered the first surveillance colonoscopy within 6 months as the index colonoscopy in these patients. Third, the surveillance intervals of the included patients were not standardized given the retrospective nature of this study. Future studies should be performed to determine the optimal surveillance schedule with comprehensive consideration of risk factors.

## Conclusion

In conclusion, dMMR status, synchronous adenoma as well as its location, longer surveillance period and times help to identify individuals at increased risk of metachronous neoplasms in patients after rectal cancer curative resection. An intensive colonoscopy surveillance strategy may be considered in these patients. Future study should be performed to determine the optimal surveillance schedule in patients after rectal cancer curative resection.

## Data Availability

The original contributions presented in the study are included in the article/Supplementary Material, further inquiries can be directed to the corresponding authors.
